# Genetic epidemiology of inherited retinal diseases in a large patient cohort followed at a single center in Italy

**DOI:** 10.1038/s41598-022-24636-1

**Published:** 2022-12-02

**Authors:** Marianthi Karali, Francesco Testa, Valentina Di Iorio, Annalaura Torella, Roberta Zeuli, Margherita Scarpato, Francesca Romano, Maria Elena Onore, Mariateresa Pizzo, Paolo Melillo, Raffaella Brunetti-Pierri, Ilaria Passerini, Elisabetta Pelo, Frans P. M. Cremers, Gabriella Esposito, Vincenzo Nigro, Francesca Simonelli, Sandro Banfi

**Affiliations:** 1 Medical Genetics, Department of Precision Medicine, Università degli Studi della Campania ’Luigi Vanvitelli’, Via Luigi De Crecchio 7, 80138 Naples, Italy; 2 Multidisciplinary Department of Medical, Surgical and Dental Sciences, Eye Clinic, Università degli Studi della Campania ’Luigi Vanvitelli’, Via Pansini 5, 80131 Naples, Italy; 3 Telethon Institute of Genetics and Medicine, Via Campi Flegrei 34, 80078 Pozzuoli, Italy; 4 Department of Genetic Diagnosis, Careggi Teaching Hospital, Florence, Italy; 5 Department of Human Genetics, Radboud University Medical Center, Nijmegen, The Netherlands; 6 Department of Molecular Medicine and Medical Biotechnologies, University of Naples Federico II, Via Pansini 5, 80131 Naples, Italy; 7 CEINGE-Advanced Biotechnologies, Via G. Salvatore 486, 80145 Naples, Italy

**Keywords:** Retinal diseases, Hereditary eye disease, Medical genetics, Disease genetics, Genetic testing, Disease genetics, Next-generation sequencing

## Abstract

Inherited retinal diseases (IRDs) are the leading cause of vision loss in the working-age population. We performed a retrospective epidemiological study to determine the genetic basis of IRDs in a large Italian cohort (n = 2790) followed at a single referral center. We provided, mainly by next generation sequencing, potentially conclusive molecular diagnosis for 2036 patients (from 1683 unrelated families). We identified a total of 1319 causative sequence variations in 132 genes, including 353 novel variants, and 866 possibly actionable genotypes for therapeutic approaches. *ABCA4* was the most frequently mutated gene (n = 535; 26.3% of solved cases), followed by *USH2A* (n = 228; 11.2%) and *RPGR* (n = 102; 5.01%). The other 129 genes had a lower contribution to IRD pathogenesis (e.g. *CHM* 3.5%, *RHO* 3.5%; *MYO7A* 3.4%; *CRB1* 2.7%; *RPE65* 2%, *RP1* 1.8%; *GUCY2D* 1.7%). Seventy-eight genes were mutated in five patients or less. Mitochondrial DNA variants were responsible for 2.1% of cases. Our analysis confirms the complex genetic etiology of IRDs and reveals the high prevalence of *ABCA4* and *USH2A* mutations. This study also uncovers genetic associations with a spectrum of clinical subgroups and highlights a valuable number of cases potentially eligible for clinical trials and, ultimately, for molecular therapies.

## Introduction

Inherited retinal diseases (IRDs) constitute a large group of rare monogenic diseases that affect primarily the retina resulting in vision impairment, and often ultimately blindness. They collectively represent the leading cause of vision loss in the working-age population with a combined incidence of 1:3000^1^. The genetic etiology of IRDs is highly heterogeneous. In most cases, IRDs follow simple inheritance patterns (autosomal dominant, autosomal recessive, X-linked and mitochondrial) and are associated with mutations in 280 genes (RetNet, http://sph.uth.edu/retnet/; accessed in April 2022). These genes encode proteins with diverse functions in the context of the retina, which range from structural components of retinal cells to key elements of the phototransduction and retinoid cycle. The complex molecular basis of IRDs mirrors an equally heterogeneous range of clinical phenotypes, varying in terms of cell-type/tissue involvement, disease onset, severity, and progression. Despite their clinical variability, a common hallmark of IRDs is photoreceptor dysfunction and death that cause different degrees of vision loss. IRDs are therefore typically classified according to the primarily affected retinal cell type. On this basis, IRDs are grouped into rod-cone dystrophies, cone-rod dystrophies, or generalized photoreceptor diseases, in which rods and cones degenerate simultaneously with an involvement of the retinal pigment epithelium (RPE)^2^. A fourth group comprises the hereditary vitreoretinopathies (exudative and erosive) which are characterized by degenerative changes in the vitreous body and the retina^2^. Another group of rare retinal diseases is albinism, where there is little or no production of melanin in conjunction with characteristic ocular and visual pathway anomalies. Optic neuropathies are a distinct disease entity in which vision loss is caused by dysfunction of the optic nerve without a direct impact on retinal integrity. Finally, additional classifications consider whether vision loss appears in the context of syndromic conditions with extraretinal involvement^3^ or in non-syndromic forms, in which only the retina is affected, and can be either progressive or stationary^2^.

The extensive phenotypic overlap of IRD subtypes hinders their accurate clinical diagnosis. Genetic testing is therefore critical because it can provide differential diagnosis and improve patient management with correct prognosis, genetic counselling, and access to gene-specific therapeutic options. Nevertheless, molecular genetics alone is often not sufficient to firmly sustain a clinical hypothesis because numerous IRD genes are associated with different clinical forms^4^, underscoring the need for the combined expertise of clinicians and ophthalmic geneticists in IRD patient management. For example, establishing the clinical subtype can impact prioritization for treatment with Voretigene Neparvovec in patients with actionable genotypes^5^.

In the last two decades, the genetic diagnosis of IRDs improved greatly thanks to the unprecedented progress in the field of DNA testing and human genomics. Publicly available databases of curated genomic variants and allelic frequencies as well as pathogenicity prediction tools have empowered variant interpretation, thereby improving diagnostic rates and accuracy. Not least, sequencing costs constantly declined making genetic screening accessible to a large fraction of patients. Consequently, there has been an exponential increase in the number of molecularly characterized patients, which offered a deeper understanding of IRD etiology and unveiled useful genotype–phenotype associations.

Given their genetic heterogeneity, recent studies quantified the relative contribution of causative genes to IRD pathogenesis in different populations^6–21^. Some of the largest cohorts described were either collected through nationwide consortia^8,16,20^ or followed in a single diagnostic center^14,15,17,19^. The largest cohorts of molecularly characterized patients reported by European initiatives included a German (2158 solved cases)^19^, British (4236 solved cases)^15^ and Spanish (2100 solved families)^14^ cohort. To date, there are no reports on Italian IRD cohorts of comparable size and clinical heterogeneity. Currently, the most comprehensive study on Italian IRD patients described the molecular epidemiology in a cohort of 221 solved probands with non-syndromic retinitis pigmentosa and Usher syndrome^22^. Other studies reported even smaller cohorts with mutations in certain genes of interest^23–26^ or with specific clinical subtypes^27–40^. Here, we report on a much larger cohort of patients which comprises a broad spectrum of clinical subgroups of inherited retinal and vitreoretinal diseases followed at a single reference center for IRDs in Italy. We collected, reviewed, and analyzed the clinical and genetic data of 2790 patients who underwent genetic testing, and identified a total of 2036 cases (73%) with a potentially conclusive molecular diagnosis. The clinical subtypes of the cohort spanned the entire spectrum of rod-dominated, cone-dominated, generalized photoreceptor or vitreoretinal degenerations, in the context of both isolated and syndromic forms. Optic neuropathies and albinism were also included. We report on the contribution of 132 genes to IRD pathogenesis and on the prevalence of 1319 distinct pathogenic alleles in the analyzed cohort. We also describe 353 novel variants in 96 genes and discuss genetic associations with clinical forms. Understanding the prevalence of causal gene defects in the overall population not only reveals the complex genetic basis of IRDs but also drives the development of targeted therapies that could be of benefit for many patients.

## Results

### Clinical composition of the genetically solved cohort

**Figure 1:**
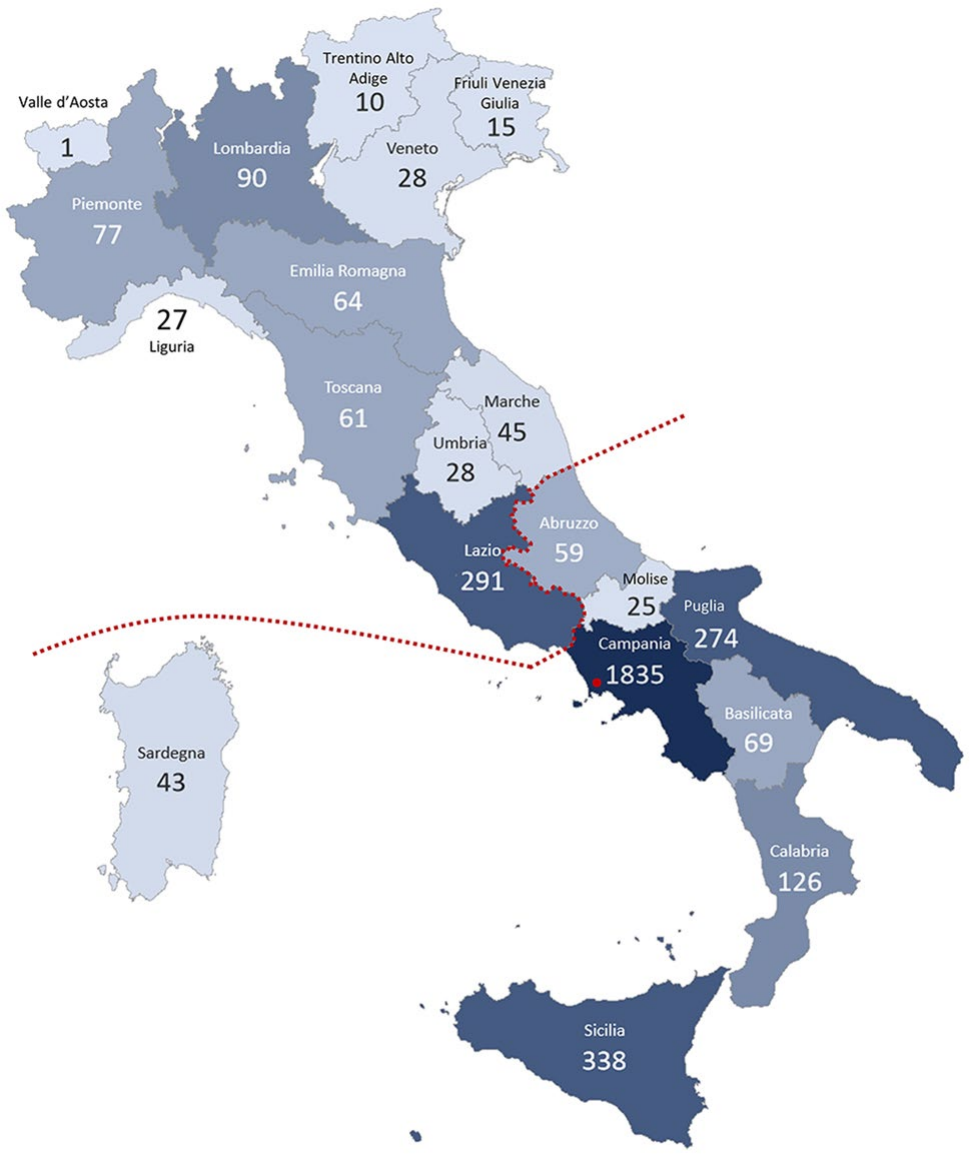
Overview of the geographical representation of the clinic’s cohort. Map of Italy, divided by administrative districts (Regions; delimited by a grey line), depicting the origin of patients followed at the Reference Center for Rare Eye Diseases at the Eye Clinic of the University of Campania ‘Luigi Vanvitelli’ (Naples, Campania Region; red dot). Color intensity indicates the relative number of patients originating from each administrative region. The geographic area corresponding to the Southern Italy and Islands is delimited by a dotted line.

The Eye Clinic of the University of Campania ‘Luigi Vanvitelli’ (Naples, Italy) is a Reference Center for rare eye diseases in Southern Italy, founded in 1991, and follows one of the largest IRD cohorts in the country, with 3514 patients enrolled in the Italian Registry of Rare Disease. Patients from all Italian regions (Fig. 1) and with clinical forms that cover the entire spectrum of IRD subtypes are referred to the clinic. Considering that Italy has 59.6 million inhabitants (census 2020) and IRDs have an overall prevalence of 1:3000, we would expect that there are 19,866 IRD patients in the Italian population. In this case, the clinic’s cohort would represent about 17.7% of the IRD patient population in Italy. By considering only the geographic area in which the Center is located (Southern Italy and Islands, resident population of 20.2 million), the clinic’s cohort (2769 patients from Southern Italy and Islands) comprises roughly 41% of the estimated IRD patients. Therefore, this cohort represents well the clinical and genetic variation of IRD patients in Italy.

In this study, we focused our attention on all patients who underwent genetic testing (n = 2790), regardless of their clinical diagnosis and their region of origin, in order to obtain a representative overview of the genetic etiology of IRDs in Italy. We examined the clinical records and genetic data of the selected patients and identified 2036 subjects (from 1683 families) with a potentially conclusive or (very) likely conclusive molecular diagnosis.

The age of the genetically defined cohort ranged from 1 to 89 years. Males and females were almost equally represented among the solved cases (55.6% vs. 44.4%). The slightly higher number of male patients is attributed to recessive X-linked conditions and the early single-gene testing of forms with a well-identifiable phenotype (e.g. retinoschisis, advanced choroideremia). Indeed, by removing the cases due to variants in X-linked genes, the sex balance was 50.17% males vs 49.83% females. The genetically solved cases comprised 1718 patients with isolated and 318 patients with syndromic forms of IRDs (84.4% and 15.6%, respectively) (Table 1). The most frequent isolated retinopathies were retinitis pigmentosa (RP; n = 733 [36%]), Stargardt disease (STGD; n = 500 [24.6%]), Leber congenital amaurosis/early-onset retinitis pigmentosa (LCA/EORP; n = 138 [6.8%]), cone/cone-rod dystrophy (CD/CRD; n = 80 [3.9%]), choroideremia (CHM; n = 68 [3.3%]), retinoschisis (RS; n = 29 [1.4%]), Best-type macular dystrophy (BEST; n = 27 [1.3%]), and achromatopsia (ACHM; n = 18 [0.9%]) (Table 1). Among the syndromic phenotypes, Usher syndrome was the most common (n = 250, representing 78.6% of syndromic forms and 12.3% of the overall cohort) (Table 1). Finally, vitreoretinal diseases (i.e. enhanced S-cone syndrome, exudative vitreoretinopathy), optic neuropathies (i.e. optic atrophy, LHON) and albinism (i.e. ocular and oculocutaneous albinism) accounted, respectively, for 0.5%, 2.8%, and 1.2% of the genetically solved cohort (Table 1).

**Table 1:** Clinical composition of the molecularly diagnosed cohort.

Clinical subtype	Genetically tested cases (n)	Solved cases (n)	Solved fraction of each clinical subtype^$^ (%)	Fraction of the entire solved cohort^‡^ (%)
Achromatopsia (ACHM)	20	18	90	0.88
Albinism	33	25	75.76	1.23
Best-type/Vitelliform macular dystrophy	36	27	75	1.33
Bietti crystalline corneoretinal dystrophy	10	7	70	0.34
Choroideremia (CHM)	68	68	100	3.34
Cone/Cone-rod dystrophy (CD/CRD)	109	80	73.40	3.93
Congenital stationary night blindness (CSNB)	15	12	80	0.59
Enhanced S-cone syndrome	10	8	80	0.39
Early-onset retinitis pigmentosa (EORP)	30	15	50	0.74
Familial exudative vitreoretinopathy	4	1	25	0.05
Gyrate atrophy of choroid and retina (GACR)	2	2	100	0.10
Leber congenital amaurosis (LCA)	170	123	72.40	6.04
Leber hereditary optic neuropathy (LHON)	51	39	76.50	1.92
Optic atrophy	48	17	35.40	0.83
Pattern dystrophy	43	13	30.20	0.64
Retinitis pigmentosa (RP)	1135	733	64.60	36.00
Retinoschisis (RS)	29	29	100	1.42
Stargardt disease (STGD)	582	500	85.90	24.56
Syndromic (Bardet-Biedl syndrome; BBS)	35	29	82.90	1.42
Syndromic (Usher syndrome type I; USHI)	91	86	94.50	4.22
Syndromic (Usher syndrome type II; USHII)	205	161	78.50	7.91
Syndromic (Usher syndrome type III; USHIII)	3	3	100	0.15
Syndromic (others) *	61	40	65.60	1.97

^$^Solved fraction of genetically tested cases with the corresponding clinical subtype.

^‡^Fraction of the solved cohort (n = 2036) that belongs to each clinical subtype.

*Alström syndrome (n = 4); Batten disease (n = 2); CHARGE syndrome (n = 2); Retinal degeneration and microcephaly (n = 1); Coffin-Siris syndrome (n = 1); Jalili syndrome (n = 2); Joubert syndrome (n = 4); Knobloch syndrome (n = 5); NARP syndrome (n = 2); Norrie disease (n = 3); peroxisome biogenesis disorder (n = 1); pseudoxantoma elasticum (n = 2); Senior-Løken syndrome (n = 2); Sorsby syndrome (n = 1); spondylometaphyseal dysplasia with cone-rod dystrophy (n = 1); Stickler syndrome (n = 4); Thiamine-responsive megaloblastic anemia and sensorineural hearing loss (n = 2); other (n = 22).

### Molecular diagnosis success rate

The overall diagnostic success rate was 69.5%, when calculated on the number of probands/families, or 73% if based on individual cases (2036 solved out of 2790 molecularly analyzed cases). The diagnostic rate varied widely depending on the genotyping methodologies used and on the specificity of the clinical phenotype. Specifically, 58% of solved cases (n = 1181) received a potentially conclusive genetic diagnosis after NGS-based genotyping by custom retinopathy panels, clinical exome or whole exome sequencing (WES) (Table 2). Roughly a third of the solved cohort (n = 587 [28.8%]) was analyzed by single-gene testing and mainly comprised subjects with well-defined clinical phenotypes, tightly associated to specific genes (e.g. STGD, CHM, RS). Finally, a significant proportion of the cohort (n = 201 [9.9%]) was genetically explained by segregation analysis of familiar disease-causing variants. Arrayed Primer Extension (APEX)-microarrays resolved 55 cases (2.7%) (Table 2). Sixty patients harboring structural variants (i.e. extended deletions or duplications) in *CHM*, *NMNAT1*, *NPHP1*, *PCDH15*, *PRPF31*, *RAX2*, *RP2*, *RPGR*, *USH1G*, *USH2A* and *WFS1* were solved by combining the above-mentioned approaches with multiplex ligation-dependent probe amplification (MLPA), array comparative genomic hybridization (aCGH)^41^, Sanger sequencing or in silico tools for CNV detection (e.g. CONTRA^42^, Vargenius^43^).

**Table 2:** Overview of the genetically solved cohort.

		Solved cases (n)	Solved cohort (%)
Sex	Female	904	44.40
	Male	1132	55.60
Genotyping method	NGS panel-based analysis	1181	58.01
	APEX-based microarray	55	2.70
	Single-gene testing	587	28.83
	Familiar variant analysis	201	9.87
	Other (array CGH, MLPA)	12	0.59
Zygosity	Homozygous	455 ^‡^	22.35
	Compound heterozygous	1002 ^‡^	49.21
	Hemizygous	238	11.69
	Heterozygous	283	13.90
	Heterozygous symptomatic carriers of X-linked forms	16	0.79
	Mitochondrial inheritance	42	2.06

		Number of variants	Fraction of identified variants (%)
Functional category	Missense	1808*	50.75
	Nonsense	609	17.10
	Frameshift	542	15.22
	Splicing site variant	430	12.07
	Other (CNV, deep-intronic, small in/del, start/stop-loss)	173	4.86

*ACMG classification of missense variants (according to VarSome, ClinVar, LOVD): 61% Pathogenic, 22% Likely Pathogenic, 17% VUS.

^‡^ Homozygosity and compound heterozygosity was confirmed by segregation analysis in 14.1% and 12.1% of cases, respectively.

### Molecular genetic composition of the cohort and causative gene prevalence

**Figure 2:**
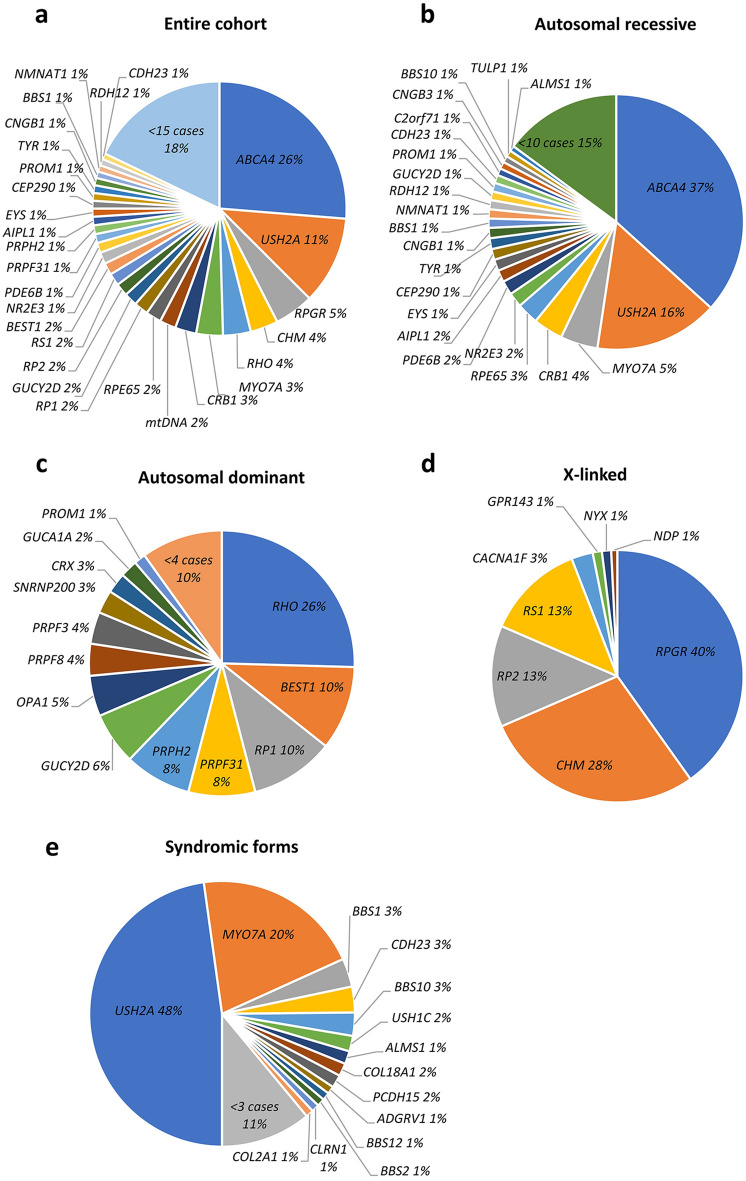
Distribution of different types of alleles in the solved cohort. (**a**) Pie chart showing the relative contribution of each causal IRD gene in disease pathogenesis of the genetically defined cohort. Genes (n = 99) implicated in less than 15 cases are plotted as a single group. (**b**–**d**) Pie chart depicting the prevalence and relative contribution of causative genes implicated in autosomal recessive (**b**), autosomal dominant (**c**) and X-linked (**d**) retinal dystrophy forms. Genes that are responsible for IRD pathogenesis in less than 10 cases with autosomal recessive (or less than 4 cases with dominant) forms are depicted as a single group. (**e**) Contribution of the most recurrent IRD-associated genes involved in syndromic forms. Genes implicated in less than 3 cases are plotted as a single group.

We identified 1319 distinct causative variants (Supplementary Table S1) in 132 different genes (Table 3). The ten most commonly mutated genes were *ABCA4* (n = 535 [26.3%])*, USH2A* (n = 228 [11.2%])*, RPGR* (n = 102 [5%]), *CHM* (n = 72 [3.5%])*, RHO* (n = 72 [3.5%])*, MYO7A* (n = 69 [3.4%])*, CRB1* (n = 55 [2.7%]), *RPE65* (n = 40 [2%]), *RP1* (n = 37 [1.8%]), and *GUCY2D* (n = 34 [1.7%]) (Table 3, Fig. 2a). The other 122 genes had a lower contribution to IRDs. One hundred genes were mutated in 15 patients or less and were collectively responsible for disease pathogenesis in 18% of the solved cohort (Fig. 2a, Table 3). Thirty-two genes were mutated in only one patient (Table 3). Mutations in the mitochondrial DNA accounted for 2.1% of the cohort and were implicated almost exclusively in the pathogenesis of LHON.

**Table 3:** Contribution of causative genes.

Gene(s)	Number of cases	Fraction of solved cohort (%)
*ABCA4*	535	26.28
*USH2A*	228	11.20
*RPGR*	102	5.01
*CHM*	72	3.54
*RHO*	72	3.54
*MYO7A*	69	3.39
*CRB1*	55	2.70
*mtDNA*	42	2.06
*RPE65*	40	1.96
*RP1*	37	1.82
*GUCY2D*	34	1.67
*RP2*	33	1.62
*RS1*	32	1.57
*BEST1*	31	1.52
*NR2E3*	30	1.47
*PDE6B*	26	1.28
*PRPF31*	23	1.13
*PRPH2*	23	1.13
*AIPL1*	22	1.08
*EYS*	21	1.03
*CEP290*	20	0.98
*PROM1*	20	0.98
*TYR*	20	0.98
*CNGB1*	19	0.93
*BBS1*	17	0.83
*NMNAT1*	17	0.83
*RDH12*	17	0.83
*CDH23*	15	0.74
*OPA1*	14	0.69
*C2orf71*	13	0.64
*CNGB3*	13	0.64
*BBS10*	12	0.59
*TULP1*	12	0.59
*PRPF3*	11	0.54
*PRPF8*	11	0.54
*SNRNP200*	11	0.54
*ALMS1*	10	0.49
*CNGA3*	9	0.44
*IMPG2, PDE6A, RPGRIP1*	8 per gene (24 in total)	0.39% per gene (1.17% in total)
*CACNA1F, CERKL, CRX, CYP4V2, GUCA1A, MERTK*	7 per gene (42 in total)	0.34% per gene (2.04% in total)
*CDHR1, USH1C*	6 per gene (12 in total)	0.29% per gene (0.58% in total)
*BBS2, COL18A1, INPP5E, PCDH15, SPATA7*	5 per gene (25 in total)	0.25% per gene (1.25% in total)
*BBS12, C8orf37, CLN3, CLRN1, GNAT2, GPR179, LRAT, PCYT1A, RLBP1*	4 per gene (36 in total)	0.20% per gene (1.77% in total)
*ADGRV1, CNGA1, CNNM4, COL2A1, FAM161A, GPR143, IFT140, IMPDH1, KCNV2, MAK, MKS1, NRL, NYX, PDE6G, RDH5*	3 per gene (45 in total)	0.15% per gene (2.21% in total)
*ABCC6, AGBL5, AHI1, BBS4, CHD7, IQCB1, KLHL7, NDP, OAT, PDE6C, PNPLA6, RAX2, RP1L1, SLC19A2, TTLL5, USH1G*	2 per gene (32 in total)	0.10% per gene (1.57% in total)
*ARSG, BBS9, C21ORF2, CACNA2D4, CEP78, EFEMP1, GRK1, HPS3, IMPG1, KCNJ13, KIAA1549, KIF11, LCA5, LRP5, MATP, MFN2, NPHP1, OTX2, PEX1, PEX10, POC1B, PRCD, PRPF6, RBP3, RD3, SMARCA4, SPG7, TMEM126A, TMEM67, TOPORS, TRPM1, TUBB4B*	1 per gene (32 in total)	0.05% per gene (1.57% in total)

**Figure 3:**
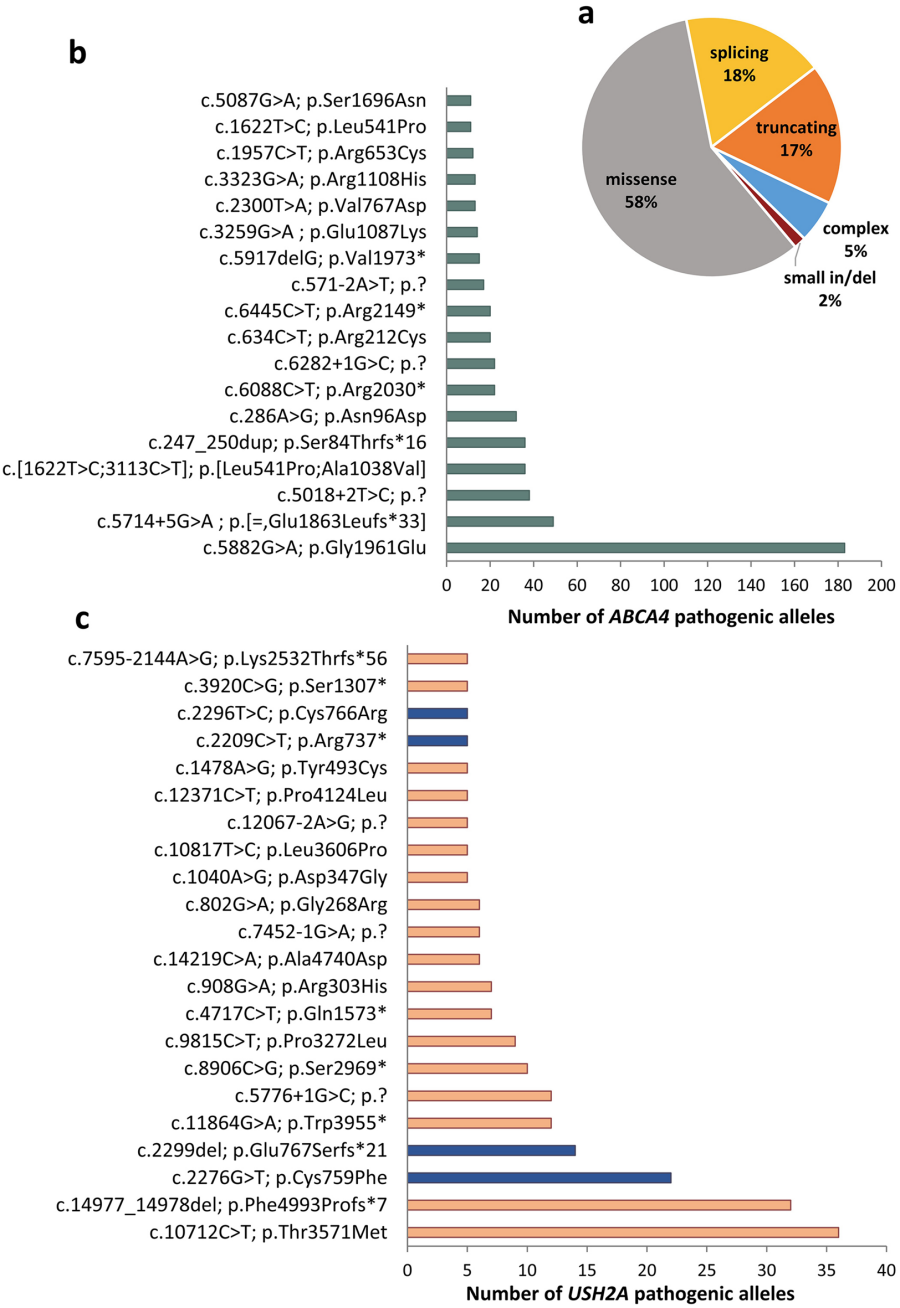
Most frequent variants identified in *ABCA4*- and *USH2A*-associated cases. (**a**) Pie-chart showing the relative abundance of the different classes of variants across the *ABCA4* pathogenic alleles. (**b**) Histogram showing the most frequent *ABCA4* alleles. (**c**) Histogram of occurrence of the most recurrent *USH2A* variants. Blue bars show variants located in exon 13.

*ABCA4* (OMIM # 601691) was the most prevalent causative gene, implicated in 535 solved cases from 453 families (Table 3; Fig. 2a). The vast majority of *ABCA4* positive cases (n = 501 [93.7%]) had a diagnosis of STGD1 (n = 470), CD/CRD (n = 28), pattern dystrophy (n = 2) or ACHM (n = 1), in line with the strong association of biallelic *ABCA4* mutations with cone-dominated phenotypes that primarily affect the central retina. Only 6.3% of *ABCA4*-associated cases (n = 34) had a diagnosis of RP (Supplementary Figure S1). Most *ABCA4*-IRD subjects (n = 477 [89.2%]) were compound heterozygous for disease-causing variants, while only 58 cases (10.8%) were homozygous. We identified 255 distinct pathogenic alleles for *ABCA4* (Supplementary Table S1). Missense variants constituted the largest fraction of the *ABCA4* disease-causing alleles (58%) followed by protein truncating and splice site variants (17.5% and 17.7%, respectively), while complex alleles were 5.3% (Fig. 3a). The contribution of the different allele types was consistent with their distribution in large collections of *ABCA4* variants^44^. The frequent hypomorphic variant c.5882G>A (p.Gly1961Glu) was the most prevalent *ABCA4* pathogenic allele (n = 183 [17.1%]) (Fig. 3b) and the most frequent variant overall in the analysed cohort (Table 4). This is the major disease-causing variant in STGD1 and is typically implicated in mild clinical phenotypes^39,44^. The second most frequent *ABCA4* variation (n = 49 alleles [4.6%]) was c.5714+5G>A (p.[= ,Glu1863Leufs*33]), classified as a moderately severe causal allele^45,46^, followed by the c.5018+2T>C (p.?) splice variant (n = 38 alleles [3.6%]) and the deleterious c.[1622T>C;3113C>T] (p.[Leu541Pro;Ala1038Val]) complex allele (n = 36 alleles [3.4%]). The c.247_250dup (p.Ser84Thrfs*16) and c.286A>G (p.Asn96Asp) variants constituted respectively 3.4% (n = 36) and 3.0% (n = 32) of the identified *ABCA4* alleles (Fig. 3a). Sixty STGD1 cases were solved using single molecule Molecular Inversion Probes (smMIPs)-based analysis of the entire *ABCA4* gene^47^, and comprised 17 patients who were initially monoallelic for *bona-fide* pathogenic variants in *ABCA4* after a first-level genetic testing (mostly carried out by Sanger sequencing or massive parallel sequencing of *ABCA4* exons).

**Table 4:** Most frequent variants in the cohort (found on at least 12 alleles).

Number of alleles	Gene	RefSeq	Genomic coordinates (hg19)	Nucleotide change	Protein change	Functional class
183	*ABCA4*	NM_000350	1:g.94473807	c.5882G>A	p.(Gly1961Glu)	Missense
49	*ABCA4*	NM_000350	1:g.94476351	c.5714+5G>A	p.[(= , Glu1863Leufs*33)]	Splicing site
38	*ABCA4*	NM_000350	1:g.94486794	c.5018+2T>C	p.(?)	Splicing site
36	*ABCA4*	NM_000350	1:g.[94528806;94508969]	c.[1622T>C;3113C>T]	p.[(Leu541Pro;Ala1038Val)]	Missense; missense
36	*ABCA4*	NM_000350	1:g.94577046_94577049	c.247_250dup	p.(Ser84Thrfs*16)	Frameshift
36	*USH2A*	NM_206933	1:g.215955412	c.10712C>T	p.(Thr3571Met)	Missense
34	*AIPL1*	NM_014336	17:g.6329101	c.834G>A	p.(Trp278*)	Nonsense
32	*ABCA4*	NM_000350	1:g.94577010	c.286A>G	p.(Asn96Asp)	Missense
32	*USH2A*	NM_206933	1:g.215812571_215812572	c.14977_14978del	p.(Phe4993Profs*7)	Frameshift
30	*MYO7A*	NM_000260	11:g.76868036	c.721C>G	p.(Arg241Gly)	Missense
22	*ABCA4*	NM_000350	1:g.94471056	c.6088C>T	p.(Arg2030*)	Nonsense
22	*ABCA4*	NM_000350	1:g.94467413	c.6282+1G>C	p.(?)	Splicing site
22	*MT-ND4*	NC_012920	m.11778	m.11778G>A	p.(Arg340His)	Missense
22	*USH2A*	NM_206933	1:g.216420460	c.2276G>T	p.(Cys759Phe)	Missense
21	*NR2E3*	NM_014249	15:g.72103821	c.119-2A>C	p. (?)	Splicing site
20	*ABCA4*	NM_000350	1:g.94564484	c.634C>T	p.(Arg212Cys)	Missense
20	*ABCA4*	NM_000350	1:g.94466426	c.6445C>T	p.(Arg2149*)	Nonsense
20	*CNGB3*	NM_019098	8:g.87656009	c.1148del	p.(Thr383Ilefs*13)	Frameshift
17	*ABCA4*	NM_000350	1:g.94564549	c.571-2A>T	p.(?)	Splicing site
17	*BBS1*	NM_024649	11:g.66293652	c.1169T>G	p.(Met390Arg)	Missense
17	*RHO*	NM_000539	3:g.129249830	c.473C>A	p.(Ala158Asp)	Missense
15	*ABCA4*	NM_000350	1:g.94473277	c.5917del	p.(Val1973*)	Nonsense
14	*ABCA4*	NM_000350	1:g.94508386	c.3259G>A	p.(Glu1087Lys)	Missense
14	*RHO*	NM_000539	3:g.129249760	c.403C>T	p.(Arg135Trp)	Missense
14	*USH2A*	NM_206933	1:g.216420436	c.2299del	p.(Glu767Serfs*21)	Frameshift
13	*ABCA4*	NM_000350	1:g.94522239	c.2300T>A	p.(Val767Asp)	Missense
13	*ABCA4*	NM_000350	1:g.94508322	c.3323G>A	p.(Arg1108His)	Missense
13	*RP1*	NM_006269	8:g.55538661	c.2219C>G	p.(Ser740*)	Nonsense
12	*ABCA4*	NM_000350	1:g.94526296	c.1957C>T	p.(Arg653Cys)	Missense
12	*CHM*	NM_000390	X:g.85211355	c.969T>A	p.(Tyr323*)	Nonsense
12	*CRB1*	NM_201253	1:g.197237598	c.57dup	p.(Ile20Tyrfs*10)	Frameshift
12	*GUCY2D*	NM_000180	17:g.7918018	c.2512C>T	p.(Arg838Cys)	Missense
12	*RP1*	NM_006269	8:g.55537407	c.965C>A	p.(Ser322*)	Nonsense
12	*RPGR*	NM_001034853	X:g.38145846_38145847	c.2405_2406del	p.(Glu802Glyfs*32)	Frameshift
12	*USH2A*	NM_206933	1:g.215901574	c.11864G>A	p.(Trp3955*)	Nonsense
12	*USH2A*	NM_206933	1:g.216246438	c.5776+1G>C	p.(?)	Splicing site

*USH2A* (OMIM # 608400) was the second most recurrent disease-causing gene, with causative variants identified in 228 subjects (from 200 families) with rod-dominated phenotypes, both syndromic and isolated (Table 3; Fig. 2a). Specifically, 67% of the patients carrying pathogenic variants in *USH2A* presented syndromic RP with hearing impairment (Usher syndrome type II) whereas 33% had isolated RP, frequently mild forms with benign prognosis such as the pericentral RP subgroup^29^ (Supplementary Figure S1). Overall, 94.4% of patients initially diagnosed with Usher type II were found to harbor mutations in *USH2A*, confirming that variants in this gene are the major cause of this condition^48^. The majority of *USH2A* genotypes were compound heterozygous (n = 172 [75.4%] vs n = 56 [24.6%] homozygotes). We identified 164 distinct variants in *USH2A,* including five pathogenic alleles harboring extended deletions (Supplementary Table S1). The largest fraction of *USH2A* pathogenic alleles (46.9%) were missense variants, while protein truncating and splicing variants constituted 43.7% and 9.6%, respectively. The most frequent variant (8.1%) was c.10712C>T (p.Thr3571Met) (Supplementary Table S1, Fig. 3b, Table 4). Nine different variants located in exon 13 were identified in 45 subjects; they represented 11.7% of all *USH2A* pathogenic alleles, with c.2276G>T (p.Cys759Phe) and c.2299del (p.Glu767Serfs*21) being the most prevalent mutations in exon 13 (Fig. 3c). Mutations within this exon are currently the target of antisense oligonucleotide-based therapies, therefore these results constitute an important framework for the future application of such approaches^49^.

Variants in the retinitis pigmentosa GTPase regulator (*RPGR*) (OMIM # 312610) were the third most frequent genetic cause in the solved cohort (Table 3; Fig. 2a). Mutations in *RPGR* were implicated in disease pathogenesis in 102 subjects (from 74 families), mostly males with X-linked RP (n = 98 [96.1%]) and less frequently (n = 4 [3.9%]) X-linked cone-dominated forms (Supplementary Figure S1). Mutations in *RPGR* were the causative genetic defect in seven female carriers who presented milder or comparable retinal disease to that encountered in the affected males of the pedigree. Roughly 60% of the disease-causing mutations in *RPGR* (mainly small deletions or duplications) occurred in the terminal exon (open reading frame 15; ORF15) of the *RPGR*^ORF15^ isoform, a mutational hotspot due to its repetitive sequence with a high GC content (Supplementary Table S1).

When considering syndromic forms with extraocular manifestations, 40 genes were identified across the 21 distinct syndromic phenotypes (Table 1). Mutations in *USH2A* and *MYO7A* were responsible for two-thirds of solved cases (48% and 20%, respectively) (Fig. 2e), in line with the high prevalence of Usher syndrome patients (78.6% of syndromic cases) (Table 1). Mutations in BBS genes (*BBS1*, *BBS10*, *BBS12*, *BBS2*, *BBS4*, *BBS9*) identified in Bardet-Biedl patients were the third most common genetic cause accounting for 9.1% of syndromic cases.

### Inheritance patterns, allelic heterogeneity and novel variants

Prior to genotyping, most cases were annotated as sporadic, based on pedigree information and family history due to the lack of affected family members. After genotyping, autosomal recessive (AR) inheritance turned out to be by far the most prevalent pattern among our patients, accounting for 71.5% (1457/2036) of solved cases. Specifically, almost half of the solved patients (n = 1,001 [49.2%]) were potentially compound heterozygous for causal variants in genes associated with recessive clinical phenotypes, while 22.4% (n = 456) were homozygous (Table 2). The most frequently mutated genes in AR forms were *ABCA4* and *USH2A*, accounting for more than half of AR cases, followed by *MYO7A* (n = 69), *CRB1* (n = 55) and *RPE65* (n = 40) (Fig. 2b).

Autosomal dominant (AD) forms represented 13.9% (n = 283) of solved cases and were associated with mutations in *RHO* (n = 72), *BEST1* (n = 29), *RP1* (n = 29), *PRPF31* (n = 23), *PRPH2* (n = 23), *GUCY2D* (n = 18), *OPA1* (n = 14), *PRPF8* (n = 11), *PRPF3* (n = 11), *SNRNP200* (n = 8), *CRX* (n = 7) and *GUCA1A* (n = 6). A further 20 genes were mutated in less than 6 cases, accounting for 32 patients with AD forms (Fig. 2c). Mutations in splicing factor genes (*PRPF31*, *PRPF8*, *PRPF3*, *SNRNP200, PRPF6*) accounted for 19.1% of causal alleles associated with AD phenotypes (n = 54). By segregation analysis in unaffected relatives (parents), we confirmed the incomplete penetrance of the autosomal dominant RP phenotype in family members of three apparently sporadic cases carrying *bona-fide* pathogenic variants in *PRPF31* (c.73G>T, p.Glu25*; c.549delG, p.Glu183Aspfs*15; c.615C>A, p.Tyr205*).

Twelve percent of cases (n = 238) were hemizygous for variants in genes linked to recessive X-linked phenotypes. The most recurrently implicated genes were *RPGR* (n = 102 [40%]), *CHM* (n = 72 [28%]), *RP2* (n = 33 [13%]) and *RS1* (n = 32 [13%]) (Fig. 2d). Mutations were also identified in *CACNA1F*, *GPR143*, *NDP* and *NYX* in a smaller number of cases. Interestingly, some cases with apparently autosomal dominant forms were reclassified post-genotyping as X-linked following the identification of mutations in *RPGR* and *RP2*. In these instances, female carriers of *RPGR*-/*RP2*-associated RP had an almost equally severe phenotype to that of affected males, mimicking a dominant inheritance. Finally, maternal inheritance of mitochondrial DNA variants was observed in 2.1% of the cohort and was almost exclusively associated with a diagnosis of LHON. Two sibs with RP and sensorimotor peripheral neuropathy (NARP syndrome) carried the m.8993T>G (p.Leu156Arg) substitution in subunit 6 of the mitochondrial ATPase gene (*MT-ATP6*).

Almost half of the pathogenic alleles (50.7%) identified in our cohort harbored single-nucleotide substitutions resulting in disease-causing missense mutations (Table 2). Protein truncating variants represented approximately 45% of the pathogenic alleles, and included nonsense (17%), frameshift (15%) and canonical splicing variants (12%) (Table 2). Small in/del changes and larger structural variants were encountered in ~ 4% of mutated alleles with 88 and 63 allele counts, respectively. We also identified 18 deep intronic variants in *CEP290* (c.2991+1665A>G; p.[Cys998*, =]), *USH2A* (c.7595-2144A>G; p.Lys2532Thrfs*56) and *ABCA4* (c.4539+2064C>T; p.[= , Arg1514Leufs*36]) as well as 68 complex alleles (23 distinct types), mostly associated with variants in *ABCA4.* Seven of these complex alleles were supported by segregation analysis (namely, four in *ABCA4* (c.[1622T>C;3113C>T], c.[2813T>C;3602T>G], c.[3323G>A;4297G>A], c.[5584+5G>A;4594G>T]), two in *USH2A* (c.[14204C>G;7939C>T], c.[2330G>A;5858C>G]) and one in *MYO7A* (c.[569A>G;1969A>T]); Supplementary Table S1).

We identified nine de novo events in patients with mutations in *GPR143*, *PRPF31*, *PRPF8*, *RHO*, *RPE65*, *RPGRIP1*, *SMARCA4* and *TUBB4B* (Table 5). In all these cases, the de novo pathogenic allele was not detected by segregation analysis in the parental samples. For three cases in which parents gave their consent to paternity testing, we could exclude non-paternity as a possible explanation. Moreover, we confirmed in a male patient with X-linked recessive ocular albinism that the causative variant was not present in the maternal sample.

**Table 5:** De novo variants identified in the cohort.

Clinical Diagnosis	Sex	Gene	RefSeq	Nucleotide change	Protein change	Variant zygosity	ACMG annotation^‡^
Ocular albinism	M	*GPR143*	NM_000273	c.101T>C	p.(Leu34Pro)	Hemizygous	Uncertain significance
Retinitis pigmentosa	M	*PRPF31*	NM_015629	c.1145A>G	p.(Glu382Gly)	Heterozygous	Pathogenic
Retinitis pigmentosa	F	*PRPF8*	NM_006445	c.6926A>G	p.(His2309Arg)	Heterozygous	Pathogenic
Retinitis pigmentosa	F	*PRPF8*	NM_006445	c.6977dup	p.(Tyr2326*)	Heterozygous	Pathogenic
Retinitis pigmentosa	F	*RHO*	NM_000539	c.403C>T	p.(Arg135Trp)	Heterozygous	Pathogenic
Retinitis pigmentosa	M	*RPE65*	NM_000329	c.1445A>T	p.(Asp482Val)	Compound heter.	Likely pathogenic
Leber congenital amaurosis	M	*RPGRIP1*	NM_020366	c.86-3T>G	p.(?)	Compound heter.	Likely pathogenic
Coffin-Siris syndrome	F	*SMARCA4*	NM_001128849	c.4297G>A	p.(Glu1433Lys)	Heterozygous	Uncertain significance
Usher syndrome	F	*TUBB4B*	NM_006088	c.1171C>T	p.(Arg391Cys)	Heterozygous	Uncertain significance

^‡^According to Varsome automated annotation (https://varsome.com/).

The genetic analysis of our cohort revealed 353 novel disease associated variants in 96 genes (Supplementary Table S2). These variants were not previously reported in ClinVar (http://www.ncbi.nlm.nih.gov/clinvar/) or in the Leiden Open Variation Database (LOVD; http://www.lovd.nl). To systematically assess their pathogenicity according to the ACMG guidelines, we retrieved the annotation reported in Varsome and interrogated the MutScore pathogenicity predictor^50^ for 122 novel missense alleles. The majority of DNA changes (n = 279 [80.6%]) were annotated as ‘likely pathogenic’ or ‘pathogenic’, while 19.4% were variants of uncertain significance (VUS). Moreover, the missense variants had an average Mutscore of 0.8 (84% with a MutScore > 0.6) corroborating their causative role in IRD pathogenesis (Supplementary Table S2).

## Discussion

Recent progress in genomic medicine increased our ability to identify the molecular causes of retinopathies in affected individuals leading to the systematic implementation of genetic analyses in clinical practice as part of standard diagnostic protocols. The large number of genetically diagnosed patients empowered epidemiological studies aimed at quantifying IRD gene prevalence. This is particularly relevant nowadays as gene-targeted therapies for IRD subtypes become a tangible prospect and it is crucial to delineate the cohort of eligible cases. Such information not only impacts assessments on disease management, prognosis and treatment options, but can also stimulate the investment of resources on therapeutic approaches that can be of benefit in large numbers of patients.

Previously, several studies have described the genetic composition of extensive nationwide or monocentric IRD cohorts from different countries^6–22,51^. These studies allow comparisons of causal variant prevalence among different populations and uncover population-specific genetic features of IRD pathogenesis. Here, we investigated the genetic features of IRDs in 2036 genetically defined subjects followed at a single referral center in Italy. As in other studies^19^, there was a prevalence of patients from the district where the Reference Center is located (i.e. Campania region, approx. 52% of the cohort) and the surrounding area (78.8% of the cohort resides in Southern Italy with a population of 20.2 million). The remaining part originated from the entire Italian territory (Fig. 1). Therefore, the clinic’s cohort can provide a representative and useful insight into the prevalence of clinical subtypes and genetic etiology in the Italian IRD community.

The genetic structure of the modern Italian population has been shaped by a series of historic migration events that induced recent demographic reshuffles and gene flow. Despite its markedly heterogeneous genomic background, Italy still has some genetic isolates in geographically secluded areas^52^. In our cohort, we identified variants that were recurrent in small, restricted local communities, suggesting a higher inbreeding. For instance, a 2.9 kb deletion in *RAX2* (19:g.3771337_3774298del), which was initially identified in a female RP patient (homozygous for this CNV)^53^, was detected also in an unrelated male patient (compound heterozygous) originating from the same small village. Another example is a novel missense variant in *RHO* (NM_000539:c.473C>A; p.Ala158Asp), which was recurrent in our cohort with 17 cases from three, apparently unrelated, families from the Campania region (Southern Italy). Lastly, the nonsense variant c.2219C>G (p.Ser740*) in *RP1* was recurrent in the Sicily island, with 13 cases from 9, apparently unrelated, families. A further corroboration to the significant extent of inbreeding in our cohort is the overall prevalence of homozygous genotypes, which were identified in about one third (n = 455 [31.23%]) of patients with recessive forms.

This is the first report on an extensive Italian cohort of comparable size and scope as other population-based reports. In terms of number of genetically characterized cases and approach, our study is similar to the German cohort described by Weisschuh et al.^19^ which comprised 1528 individuals with a conclusive genetic analysis followed at a single diagnostic center. Our cohort is also comparable in size and spectrum of clinical phenotypes with reports from Israel (1369 solved families)^16^, Spain (2100 families)^14^ and North America (760 solved families)^17^. The genetic composition of our solved cohort was equally complex compared to that of other reports, with a total of 132 genes implicated in the pathogenesis of 42 clinical subtypes. The relative contribution of causative genes was also largely in line. When considering all clinical subtypes, the three most frequently mutated genes were *ABCA4*, *USH2A* and *RPGR*, thereby confirming their high contribution to IRD pathogenesis reported in other populations^8,14–17,19^. The observed frequency of *ABCA4-* and *USH2A-*associated genotypes was consistent with the high prevalence of variants in these genes in at least five main world populations^54^. Variants in *CHM* were the fourth most common genetic IRD cause in our cohort, yet their prevalence could be in part skewed by the easily identifiable clinical phenotype of advanced forms and single-gene etiology of CHM which enabled early candidate gene analyses and high diagnostic rates for affected subjects (Table 1). The same possibly applies to *RS1* and *BEST1* that ranked, respectively, at position 13 and 14 (Tables 1, 2).

Rod-dominated phenotypes had the highest genetic heterogeneity, with 75 causal genes explaining disease etiology in 733 patients with RP, which represented the most common subtype. Comparatively, a smaller number of genes was associated with cone-dominated phenotypes, with the extreme example of recessive STGD forms, which were almost exclusively caused by mutations in *ABCA4*. Overall, our findings suggest that the genetic causes underlying IRD pathogenesis in Italy are mostly in line with those reported for other cohorts. Differences in the relative causal gene frequencies with other populations could be attributed to local founder mutations or to the specialized clinical focus of each diagnostic center. For example, variants in *FAM161A* were detected only in 3 cases, while it was the third most common mutated gene in Israel due to founder mutations^16^. An uninvestigated founder effect may also explain the high prevalence of *CYP4V2* mutations (c.802-8_810delinsGC) in Taiwan^51^ and China^55^. Other discrepancies in observed genetic variant frequencies could be due to small cohort sizes, e.g. comparatively high frequency of *RLBP1* variants in Iceland^18^.

Compared to a study on an Italian cohort of 221 molecularly diagnosed cases^22^, we herein report a larger cohort of 2036 solved cases and include a spectrum of clinical entities that extends beyond non-syndromic RP and Usher syndrome. Specifically, our study offers an overview of the pathogenesis of macular/cone dominated conditions as roughly a third of the cohort (n = 625) had such phenotypes. To date, the largest Italian cohort of genetically defined patients with macular and cone/cone-rod dystrophy comprised 136 cases^37^. Moreover, we describe genetically solved cases with a diagnosis of CHM, RS, optic atrophy, LHON, vitreoretinopathies and albinism as well as a spectrum of syndromic forms which, besides Usher, comprise BBS, JBS, Alström and Knobloch phenotypes among others. Regarding the clinical phenotypes common to both studies (i.e. non-syndromic RP and Usher syndrome^22^, and macular, cone/cone-rod dystrophies^37^), we find extensive overlap of genetic causes, as expected.

When compiling our study cohort, we recruited patients with rare monogenic eye diseases that cause visual impairment, even when these did not strictly fit in the IRD classification proposed by Berger^2^. Specifically, we also recruited patients with optic neuropathies (including LHON) and albinism in order to understand disease and mutation prevalence, given that these patients are normally referred to the Rare Ocular Disease units and pharmacological treatment options (e.g. idebenone) are available. As expected, a diagnosis of LHON was almost exclusively associated with the three common mtDNA mutations m.11778G>A, m.3460G>A and m.14484T>C (relative frequency of 52%, 26% and 5%, respectively).

Mutations in *RPGR* were the third most common cause of IRDs, accounting for 5.2% of our genetically diagnosed patients (n = 102 cases of *RPGR*-associated IRD), as also observed in other populations^14,19,56^. The contribution of *RPGR* to IRD pathogenesis is likely underestimated since about 60% of the disease-causing mutations are located in the terminal exon (open reading frame 15; ORF15) of the *RPGR*^*ORF15*^ isoform which is refractory to NGS-based analyses due to its repetitive, purine-rich sequence. The insufficient coverage of ORF15 in NGS experiments warrants the implementation of complementary approaches to probe this mutational hot-spot, especially in males with a negative NGS analysis, compatible clinical presentation (RP or CD) and/or evidence of X-linked transmission, considering both the high incidence of *RPGR* mutations as well as the concrete therapeutic perspectives for *RPGR*-associated forms (e.g. ClinicalTrials.gov Identifier: NCT03252847).

Segregation analysis of the identified variants was performed in 22.3% of the cases, as in the case of other studies of similar magnitude. Sample unavailability did not allow us to systematically confirm biallelism, especially for genes with frequent complex alleles, such as *ABCA4*, in which certain variants (e.g. c.1622T>C, c.3113C>T) have been described both as part of complex and simple alleles^44^.

Herein, we give a detailed account of the allelic heterogeneity in genetically defined IRD patients from a large Italian cohort and report over 300 novel variants, not previously described, to the best of our knowledge. To establish whether a genotype could explain the disease, we assessed the concordance with the inheritance mode, available segregation results and, most importantly, the clinical phenotype. A total of 15 cases (out of 2036) were clinically reconsidered and reclassified after genetic testing: 6 cases with a non-RP diagnosis and mutations in *ABCA4, CRB1*, *NRL*, *RLBP1, PRPF6*, *RPGR* were reclassified as RP, whereas 9 RP cases were revised as non-RP (3 cases with mutations in *NR2E3* and *CHM*) or as syndromic IRD forms (6 cases with mutations in *SMARCA4*, *AHI1*, *CLN3, PCYT1A, MFN2* and *MKS1*^57^). Therefore, the integrative management of IRD patients should avail the combined expertise of clinicians and ophthalmic geneticists.

Overall, we identified 866 patients (42.5% of the solved cohort) with potentially actionable genotypes for therapeutic approaches (both pharmacological and gene therapy-based) that are either already available [e.g. Luxturna for *RPE65* (n = 40), idebenone for mtDNA mutations causing LHON (n = 41)] or are currently being tested in advanced clinical trials (Phase II/III) [e.g. *ABCA4* (n = 535), *CHM* (n = 72)*, CEP290:*c.2991+1665A>G (n = 7), *CNGA3* (n = 9)*, MERTK* (n = 7), *PDE6A* (n = 8)*, RPGR* (n = 102), *USH2A*-exon13 (n = 45)]. We expect that the number of molecularly diagnosed patients will increase further thanks to the availability of gene-specific treatments that stimulate patients to actively seek genetic diagnosis. Defining the molecular epidemiology of IRDs, besides providing insights on their molecular etiology, can advise policymakers and stakeholders on the healthcare burden of these rare diseases, and can act as a driver to guide research efforts on the development of therapeutic options for a growing number of patients.

## Methods

### Patient selection and ethical statement

We reviewed 2790 patients with IRD based on clinical records (i.e. clinical diagnosis, family history, clinical history, systemic findings, visual acuity tests, fundus changes, visual field assessment, optical coherence tomography imaging, fundus autofluorescence, electroretinography) available at the Center for Inherited Retinal Dystrophies of the Eye Clinic, University of Campania ‘Luigi Vanvitelli’. Only patients who were willing to undergo, or had already undergone, genetic screening were included in the study. Procedures adhered to the tenets of the Declaration of Helsinki and were approved by the Ethics Board of the University of Campania ‘Luigi Vanvitelli’. Informed consent to genetic testing and data sharing was obtained from the patients (or their parents/legal guardians for minors). Available reports from genetic analyses commissioned by the patient or by other medical practitioners were voluntarily provided by the patient for archiving in their medical record.

### Genotyping methods

Different genotyping methods have been used over the years, following the technical evolution of sequencing methodologies. Earlier analyses were based on single gene testing (i.e. by PCR on genomic DNA followed by Sanger sequencing) whenever the clinical phenotype and inheritance pattern were strongly indicative of a candidate gene (e.g. *ABCA4* for recessive STGD, *CHM* in choroideremia, *RS1* in retinoschisis). Later, APEX-based genotyping microarrays (www.asperbio.com; Asper Biotech, Ltd.) were used to screen for known mutations implicated in LCA, RP or STGD. Starting from 2013, samples were screened by high-throughput targeted sequencing (including smMIPs-based analysis of a single or few candidate genes e.g. *ABCA4* and *PRPH2* for STGD patients)^47^. More recently, patient samples were analysed using custom panels of known retinopathy genes^28^, clinical exome sequencing or WES (Supplementary Table S3). Some cases with a well-defined clinical diagnosis which was commonly associated with a limited number of genes (e.g. patients with Usher syndrome^48^, albinism, cone dystrophy, LCA), underwent a first-tier analysis on restricted gene panels relevant to their condition.

For some cases that remained unsolved after an NGS-based analysis, we implemented complementary approaches to identify disease-causing mutations (e.g. Sanger-based analysis of *RPGR*^*ORF15*^^23^, search for rare structural variants/larger copy number variations (CNVs) by experimental and in silico approaches^41–43^). Because whole-exome and clinical exome approaches do not efficiently detect all known deep-intronic variants associated with IRDs, we screened by Sanger sequencing known intronic variants (e.g. *CEP290*:c.2991+1655A>G, *USH2A*:c.7595-2144A>G) whenever these were not present in the panel used and patients had a compatible clinical phenotype and/or monoallelic pathogenic variants in these genes.

For all genetic analyses, DNA was extracted from peripheral blood samples using standard protocols. The identified variants were always validated by Sanger sequencing. Segregation analysis was performed whenever parental DNA (or samples from other family members) were available. For NGS analyses, library preparation, sequencing and sequence data analysis was performed as previously described^27^. Filtered reads were visually inspected on the Integrative Genomics Viewer (IGV).

### Pathogenicity assessment of sequence variants and criteria for genotype classification

The pathogenicity of sequence variants was assessed according to the guidelines of the American College of Medical Genetics and Genomics (ACMG)^58^, either by manual implementation of the criteria or using the Varsome automated variant classification^59^. For the annotation of the novel missense variants, we availed of the MutScore pathogenicity predictor^50^ to corroborate the Varsome annotation. For the scope of this study, we considered as ‘genetically (likely) solved’:

- patients carrying monoallelic ‘pathogenic’ (P) or ‘likely pathogenic’ (LP) variants in genes associated with dominant phenotypes or with recessive X-linked IRD forms (only for males);
- patients with a homozygous or two heterozygous variants (P, LP or variants of uncertain significance [VUS]) in a gene associated with recessive phenotypes consistent with their clinical presentation. We confirmed biallelism of heterozygous variants whenever segregation analysis was possible. However, since segregation analysis could not be systematically applied due to sample unavailability, we did not set it as a prerequisite for inclusion among the ‘solved’ cases, in line with studies on similar sized cohorts^19^;
- patients with *bona-fide* mutation in the mitochondrial DNA (mtDNA).

## Supplementary Information

Supplementary Information 1.

Supplementary Information 2.

## Data Availability

All data analysed during this study are included in this published article (and its Supplementary Information files). Novel variants identified in this study are currently being deposited in the Leiden Open Variation Database (LOVD) (http://www.lovd.nl/).

## References

[1] Sahel JA, Marazova K, Audo I (2014). Clinical characteristics and current therapies for inherited retinal degenerations. Cold Spring. Harb. Perspect. Med..

[2] Berger W, Kloeckener-Gruissem B, Neidhardt J (2010). The molecular basis of human retinal and vitreoretinal diseases. Prog. Retin. Eye Res..

[3] Tatour Y, Ben-Yosef T (2020). Syndromic inherited retinal diseases: Genetic, clinical and diagnostic aspects. Diagnostics (Basel).

[4] Schatz P (2011). Fundus albipunctatus associated with compound heterozygous mutations in RPE65. Ophthalmology.

[5] Aoun M (2021). Inherited retinal diseases due to RPE65 variants: From genetic diagnostic management to therapy. Int. J. Mol. Sci..

[6] Carss KJ (2017). Comprehensive rare variant analysis via whole-genome sequencing to determine the molecular pathology of inherited retinal disease. Am. J. Hum. Genet..

[7] Dockery A (2017). Target 5000: Target capture sequencing for inherited retinal degenerations. Genes.

[8] Goetz KE (2020). Genetic testing for inherited eye conditions in over 6,000 individuals through the eyeGENE network. Am. J. Med. Genet. C Semin. Med. Genet..

[9] Holtan JP, Selmer KK, Heimdal KR, Bragadóttir R (2020). Inherited retinal disease in Norway—a characterization of current clinical and genetic knowledge. Acta Ophthalmol..

[10] Jespersgaard C (2019). Molecular genetic analysis using targeted NGS analysis of 677 individuals with retinal dystrophy. Sci. Rep..

[11] Koyanagi Y (2019). Genetic characteristics of retinitis pigmentosa in 1204 Japanese patients. J. Med. Genet..

[12] Ma DJ (2021). Whole-exome sequencing in 168 Korean patients with inherited retinal degeneration. BMC Med. Genomics.

[13] Motta FL, Martin RP, Filippelli-Silva R, Salles MV, Sallum JMF (2018). Relative frequency of inherited retinal dystrophies in Brazil. Sci. Rep..

[14] Perea-Romero I (2021). Genetic landscape of 6089 inherited retinal dystrophies affected cases in Spain and their therapeutic and extended epidemiological implications. Sci. Rep..

[15] Pontikos N (2020). Genetic basis of inherited retinal disease in a molecularly characterized cohort of more than 3000 families from the United Kingdom. Ophthalmology.

[16] Sharon D (2020). A nationwide genetic analysis of inherited retinal diseases in Israel as assessed by the Israeli inherited retinal disease consortium (IIRDC). Hum. Mutat..

[17] Stone EM (2017). Clinically focused molecular investigation of 1000 consecutive families with inherited retinal disease. Ophthalmology.

[18] Thorsteinsson DA, Stefansdottir V, Eysteinsson T, Thorisdottir S, Jonsson JJ (2021). Molecular genetics of inherited retinal degenerations in Icelandic patients. Clin. Genet..

[19] Weisschuh N (2020). Genetic architecture of inherited retinal degeneration in Germany: A large cohort study from a single diagnostic center over a 9-year period. Hum. Mutat..

[20] Whelan L (2020). Findings from a genotyping study of over 1000 people with inherited retinal disorders in Ireland. Genes (Basel).

[21] Zenteno JC (2020). Extensive genic and allelic heterogeneity underlying inherited retinal dystrophies in Mexican patients molecularly analyzed by next-generation sequencing. Mol. Genet. Genomic Med..

[22] Colombo L (2021). Molecular epidemiology in 591 Italian probands with nonsyndromic retinitis pigmentosa and usher syndrome. Invest. Ophthalmol. Vis. Sci..

[23] Di Iorio V (2020). Spectrum of disease severity in patients with X-linked retinitis pigmentosa due to RPGR mutations. Invest. Ophthalmol. Vis. Sci..

[24] Testa F (2022). RPE65-associated retinopathies in the Italian population: A longitudinal natural history study. Invest. Ophthalmol. Vis. Sci..

[25] Testa F (2021). Spectrum of disease severity in nonsyndromic patients with mutations in the CEP290 gene: A multicentric longitudinal study. Invest. Ophthalmol. Vis. Sci..

[26] Mucciolo DP (2018). Fundus phenotype in retinitis pigmentosa associated with EYS mutations. Ophthalmic Genet..

[27] Brunetti-Pierri R (2021). Clinical and molecular characterization of achromatopsia patients: A longitudinal study. Int. J. Mol. Sci..

[28] Di Iorio V (2017). Clinical and genetic evaluation of a cohort of pediatric patients with severe inherited retinal dystrophies. Genes (Basel).

[29] Karali M (2019). Clinical and genetic analysis of a European cohort with pericentral retinitis pigmentosa. Int. J. Mol. Sci..

[30] Suppiej A (2021). Exome sequencing and electro-clinical features in pediatric patients with very early-onset retinal dystrophies: A cohort study. Eur. J. Paediatr. Neurol..

[31] Pierrottet CO (2014). Syndromic and non-syndromic forms of retinitis pigmentosa: A comprehensive Italian clinical and molecular study reveals new mutations. Genet. Mol. Res..

[32] Sodi A (2014). MYO7A and USH2A gene sequence variants in Italian patients with Usher syndrome. Mol. Vis..

[33] Lenarduzzi S (2019). Next generation sequencing study in a cohort of Italian patients with syndromic hearing loss. Hear Res..

[34] Ziviello C (2005). Molecular genetics of autosomal dominant retinitis pigmentosa (ADRP): A comprehensive study of 43 Italian families. J. Med. Genet..

[35] Simonelli F (2007). Clinical and molecular genetics of Leber's congenital amaurosis: a multicenter study of Italian patients. Invest Ophthalmol Vis Sci.

[36] Esposito G (2011). Comprehensive mutation analysis (20 families) of the choroideremia gene reveals a missense variant that prevents the binding of REP1 with Rab geranylgeranyl transferase. Hum. Mutat..

[37] Falsini B (2022). Genetic characteristics of 234 Italian patients with macular and cone/cone-rod dystrophy. Sci. Rep..

[38] Di Iorio V (2019). CHM/REP1 transcript expression and loss of visual function in patients affected by choroideremia. Invest. Ophthalmol. Vis. Sci..

[39] Di Iorio V (2019). Association between genotype and disease progression in Italian Stargardt patients: A retrospective natural history study. Retina.

[40] Esposito G (2017). Genetic characterization of Italian patients with Bardet-Biedl syndrome and correlation to ocular, renal and audio-vestibular phenotype: Identification of eleven novel pathogenic sequence variants. BMC Med. Genet..

[41] Fioretti T (2021). Molecular characterization of choroideremia-associated deletions reveals an unexpected regulation of CHM gene transcription. Genes (Basel).

[42] Li J (2012). CONTRA: Copy number analysis for targeted resequencing. Bioinformatics.

[43] Astuti GD (2016). Mutations in AGBL5, encoding alpha-tubulin deglutamylase, are associated with autosomal recessive retinitis pigmentosa. Invest. Ophthalmol. Vis. Sci..

[44] Cremers FPM, Lee W, Collin RWJ, Allikmets R (2020). Clinical spectrum, genetic complexity and therapeutic approaches for retinal disease caused by ABCA4 mutations. Prog. Retin. Eye Res..

[45] Lee W (2022). A genotype-phenotype correlation matrix for ABCA4 disease based on long-term prognostic outcomes. JCI Insight.

[46] Cornelis SS (2022). Personalized genetic counseling for Stargardt disease: Offspring risk estimates based on variant severity. Am. J. Hum. Genet..

[47] Khan M (2020). Resolving the dark matter of ABCA4 for 1054 Stargardt disease probands through integrated genomics and transcriptomics. Genet. Med..

[48] Bonnet C (2016). An innovative strategy for the molecular diagnosis of Usher syndrome identifies causal biallelic mutations in 93% of European patients. Eur. J. Hum. Genet..

[49] Dulla K (2021). Antisense oligonucleotide-based treatment of retinitis pigmentosa caused by USH2A exon 13 mutations. Mol. Ther..

[50] Quinodoz M (2022). Analysis of missense variants in the human genome reveals widespread gene-specific clustering and improves prediction of pathogenicity. Am. J. Hum. Genet..

[51] Chen TC (2021). Genetic characteristics and epidemiology of inherited retinal degeneration in Taiwan. NPJ Genom. Med..

[52] Capocasa M (2014). Linguistic, geographic and genetic isolation: A collaborative study of Italian populations. J. Anthropol. Sci..

[53] Van de Sompele S (2019). Biallelic sequence and structural variants in RAX2 are a novel cause for autosomal recessive inherited retinal disease. Genet. Med..

[54] Hanany M, Rivolta C, Sharon D (2020). Worldwide carrier frequency and genetic prevalence of autosomal recessive inherited retinal diseases. Proc. Natl. Acad. Sci. USA.

[55] Wang L (2018). Application of whole exome and targeted panel sequencing in the clinical molecular diagnosis of 319 Chinese families with inherited retinal dystrophy and comparison study. Genes (Basel).

[56] Shu X (2007). RPGR mutation analysis and disease: An update. Hum. Mutat..

[57] Brunetti-Pierri R (2021). Mild clinical presentation of Joubert syndrome in a male adult carrying biallelic MKS1 truncating variants. Diagnostics (Basel).

[58] Richards S (2015). Standards and guidelines for the interpretation of sequence variants: A joint consensus recommendation of the American College of Medical Genetics and Genomics and the Association for Molecular Pathology. Genet. Med..

[59] Kopanos C (2019). VarSome: The human genomic variant search engine. Bioinformatics.

