# Supplementary material for: Genetic epidemiology of inherited retinal diseases in a large patient cohort followed at a single center in Italy
Source: Sci Rep. 2022 Dec 2;12:20815. doi: 10.1038/s41598-022-24636-1 (PMC9718770; doi:10.1038/s41598-022-24636-1)
Supplement: Supplementary file 1 — Supplementary Information 1. [file 41598_2022_24636_MOESM1_ESM.pdf]

SUPPLEMENTARY INFORMATION

Genetic epidemiology of inherited retinal diseases in a large patient cohort followed at a single center in Italy

Marianthi Karali[1,2†], Francesco Testa[2†], Valentina Di Iorio[2], Annalaura Torella[1,3], Roberta Zeuli[1], Margherita Scarpato[1], Francesca Romano[1], Maria Elena Onore[1], Mariateresa Pizzo[3], Paolo Melillo[2], Raffaella Brunetti-Pierri[2], Ilaria Passerini[4], Elisabetta Pelo[4], Frans P. M. Cremers[5], Gabriella Esposito[6], Vincenzo Nigro[1,3], Francesca Simonelli[2*], Sandro Banfi[1,3*]

[1] Medical Genetics, Department of Precision Medicine, Università degli Studi della Campania 'Luigi Vanvitelli', via Luigi De Crecchio 7, Naples 80138, Italy

[2] Eye Clinic, Multidisciplinary Department of Medical, Surgical and Dental Sciences, Università degli Studi della Campania 'Luigi Vanvitelli', via Pansini 5, Naples 80131, Italy

[3] Telethon Institute of Genetics and Medicine, via Campi Flegrei 34, Pozzuoli 80078, Italy

[4] Department of Genetic Diagnosis, Careggi Teaching Hospital, Florence, Italy

[5] Department of Human Genetics, Radboud University Medical Center, Nijmegen, The Netherlands

[6] Department of Molecular Medicine and Medical Biotechnologies, University of Naples Federico II, via Pansini 5, Naples 80131, Italy; CEINGE-Advanced Biotechnologies, via G. Salvatore 486, Naples 80145, Italy

SUPPLEMENTARY FIGURE

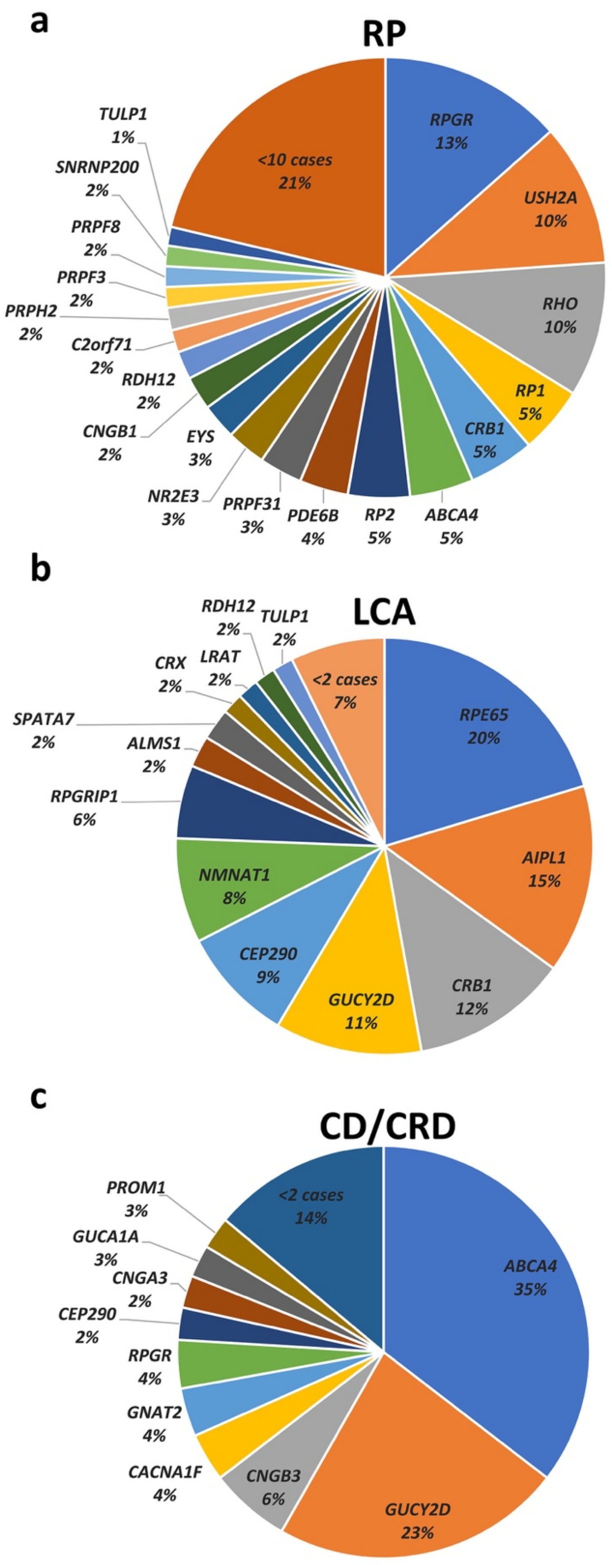

Supplementary Figure S1. Contribution of causal genes to the most common IRD subtypes. Pie charts showing the relative contribution of the main causal genes in disease pathogenesis of the RP (**a**), LCA (**b**) and CD/CRD (**c**) cohorts. Genes implicated in less than 10 RP cases or in less than 2 LCA or CD/CRD cases are grouped.

SUPPLEMENTARY TABLES

Supplementary Table S1. List of all identified variants, their frequency and associated inheritance pattern in the genetically solved cohort.

Supplementary Table S2. Novel variants identified in this study (not reported in ClinVar and LOVD).

Supplementary Table S3. *In silico* IRD gene panel used for clinical and whole exome sequencing analyses.
